# Supplementary material for: An open source automated tumor infiltrating lymphocyte algorithm for prognosis in melanoma
Source: Nat Commun. 2019 Nov 29;10:5440. doi: 10.1038/s41467-019-13043-2 (PMC6884485; doi:10.1038/s41467-019-13043-2)

Supplementary Information

An Open Source Automated Tumor Infiltrating Lymphocyte Algorithm for Prognosis in Melanoma

Acs *et* al.

List of Content

Supplementary Figure Legends

Supplementary Figures 1-3

Supplementary Tables 1-2

Supplementary Figure Legends

Supplementary Figure 1

Association between eTIL% score and the clinicopathological factors in cohort #1, #2, #3 and #4. eTIL% score was higher in cases with absent ulceration and smaller tumor depth in cohorts #1, #2 and #3. No significant association was found between eTIL% and any clinicopatholigcal factor in cohort#4.

Supplementary Figure 2

Reproducibility between corresponding TMA and WSI cases regarding eTIL% scores. Intra-class correlation coefficient (ICC).

Supplementary Figure 3

Automated TIL scoring in whole slide cohorts. In the whole slide cohorts (cohort #2 and #4), the analysis was run on the entire tumor area (A, C, D) following pathologists' markings (B). One whole slide per patient selected by a pathologist was investigated in the study.

Supplementary Figure 1

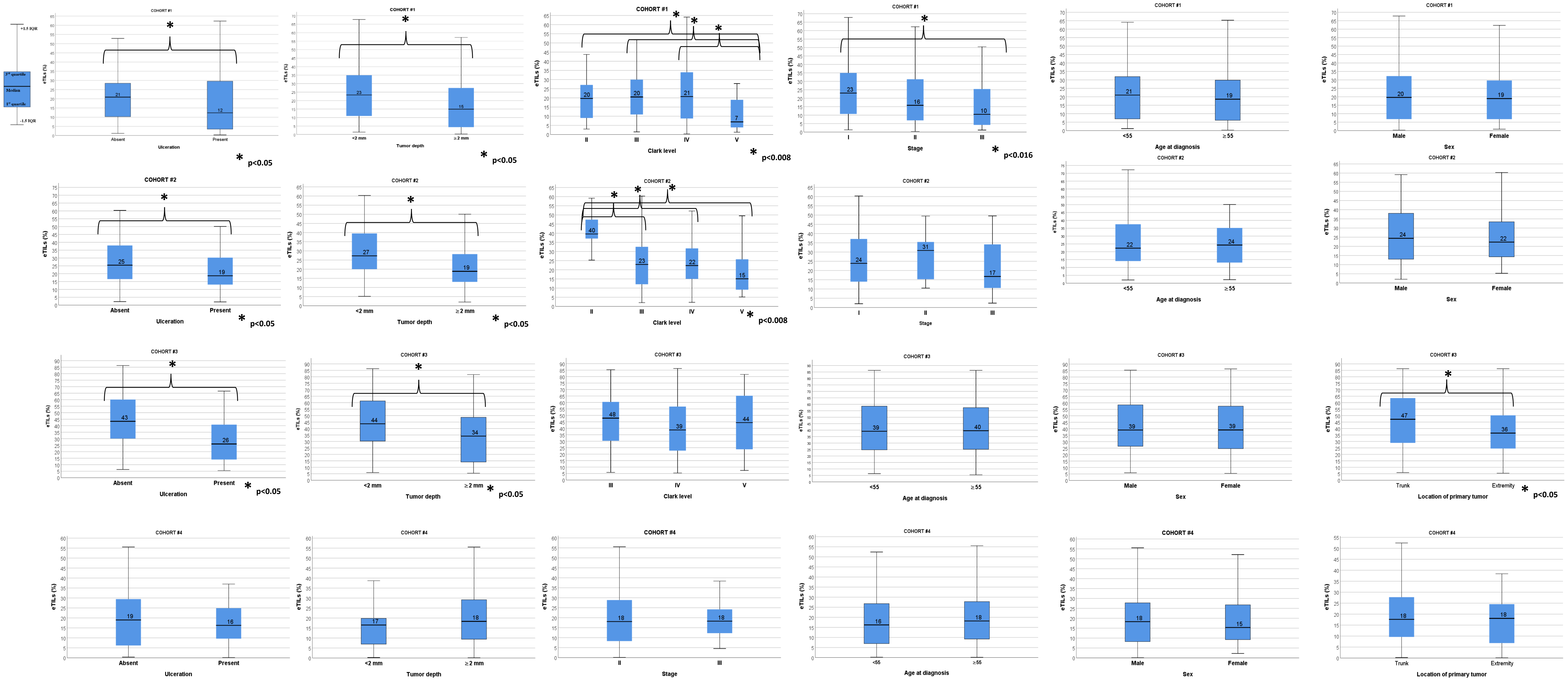

Supplementary Figure 2

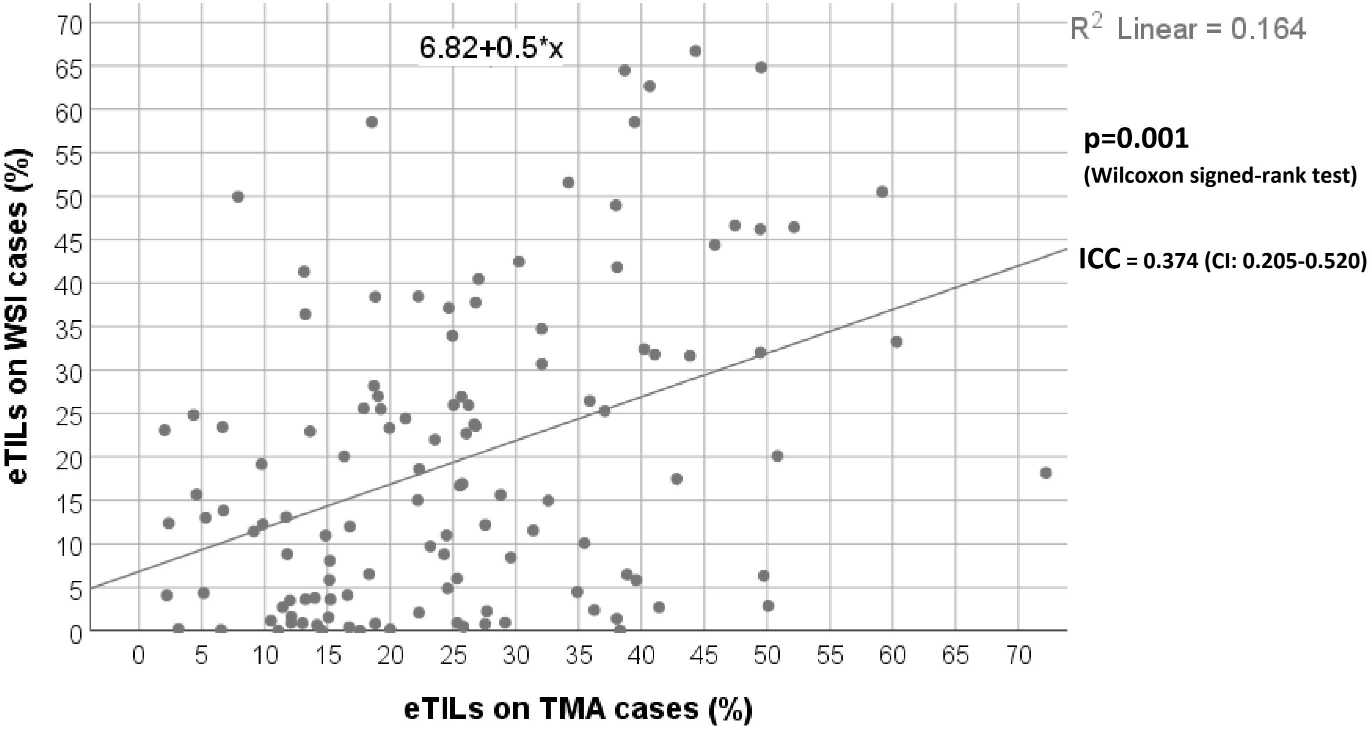

Supplementary Figure 3

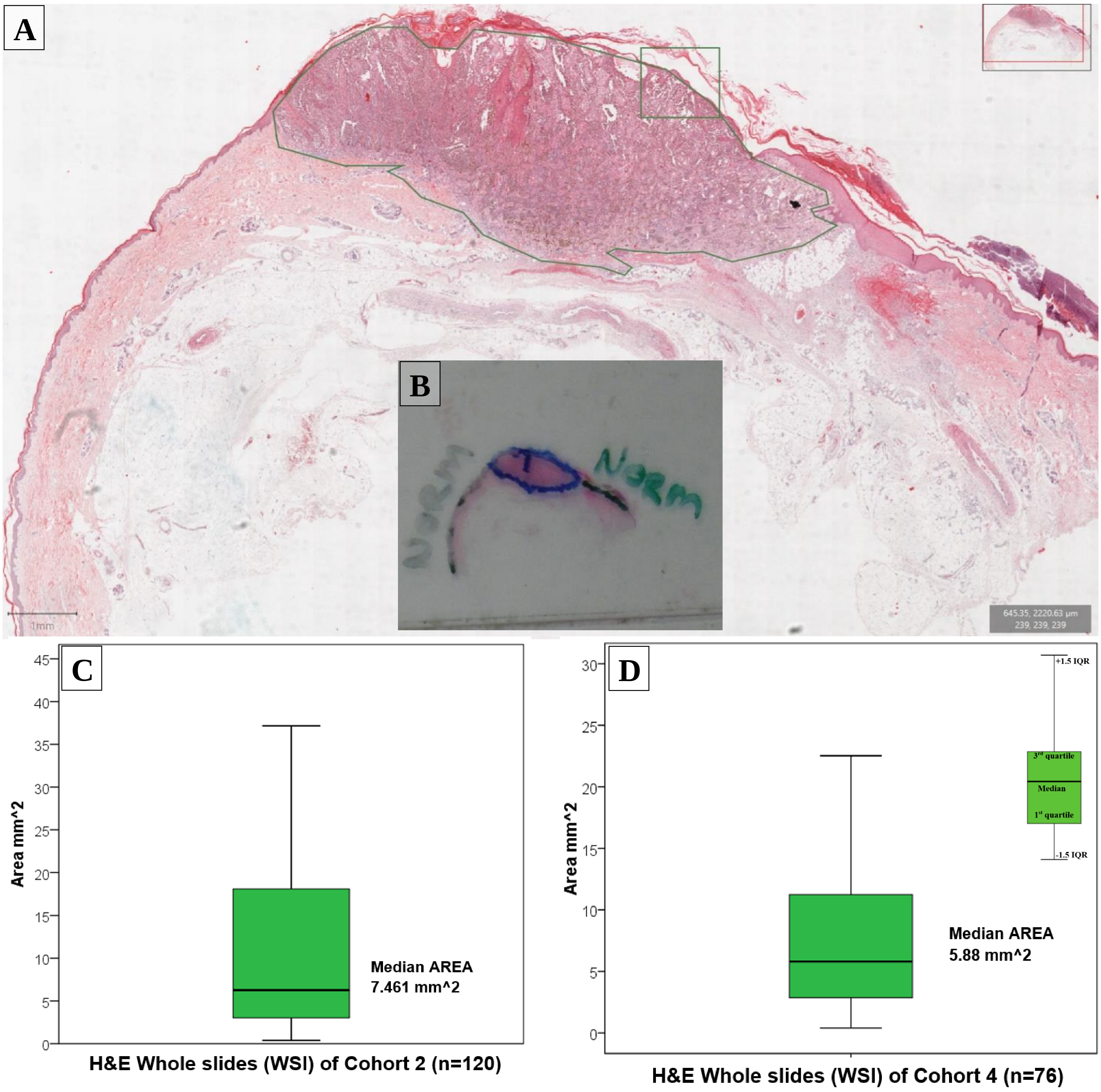

Supplement: Supplementary file 1 — Supplementary Information [file 41467_2019_13043_MOESM1_ESM.pdf]
