# Supplementary material for: An open source automated tumor infiltrating lymphocyte algorithm for prognosis in melanoma
Source: Nat Commun. 2019 Nov 29;10:5440. doi: 10.1038/s41467-019-13043-2 (PMC6884485; doi:10.1038/s41467-019-13043-2)
Supplement: Supplementary file 3 — Reporting Summary [file 41467_2019_13043_MOESM3_ESM.pdf]

nature research

Corresponding author(s): David L. Rimm, MD, PhD

Last updated by author(s): Feb 15, 2019

Reporting Summary

Nature Research wishes to improve the reproducibility of the work that we publish. This form provides structure for consistency and transparency in reporting. For further information on Nature Research policies, see Authors & Referees and the Editorial Policy Checklist .

Statistics

For all statistical analyses, confirm that the following items are present in the figure legend, table legend, main text, or Methods section.

n/a	Confirmed	
☐	☒	The exact sample size (*n*) for each experimental group/condition, given as a discrete number and unit of measurement
☐	☒	A statement on whether measurements were taken from distinct samples or whether the same sample was measured repeatedly
☐	☒	The statistical test(s) used AND whether they are one- or two-sided<br>*Only common tests should be described solely by name; describe more complex techniques in the Methods section.*
☐	☒	A description of all covariates tested
☐	☒	A description of any assumptions or corrections, such as tests of normality and adjustment for multiple comparisons
☐	☒	A full description of the statistical parameters including central tendency (e.g. means) or other basic estimates (e.g. regression coefficient) AND variation (e.g. standard deviation) or associated estimates of uncertainty (e.g. confidence intervals)
☐	☒	For null hypothesis testing, the test statistic (e.g. *F*, *t*, *r*) with confidence intervals, effect sizes, degrees of freedom and *P* value noted<br>*Give P values as exact values whenever suitable.*
☒	☐	For Bayesian analysis, information on the choice of priors and Markov chain Monte Carlo settings
☐	☒	For hierarchical and complex designs, identification of the appropriate level for tests and full reporting of outcomes
☐	☐	Estimates of effect sizes (e.g. Cohen's *d*, Pearson's *r*), indicating how they were calculated

Our web collection on statistics for biologists contains articles on many of the points above.

Software and code

Policy information about availability of computer code

Data collection	For data collection, we used an open source software called QuPath. The software and its documentation can be found at https://qupath.github.io/
Data analysis	We used watershed cell detection to segment the cells in the image with the following settings: Detection image: Hematoxylin OD; Requested pixel size: 0.5 µm; Background radius: 8 µm; Median filter radius: 0 µm; sigma: 1.5 µm; Minimum cell area: 10 µm2; Maximum cell area: 400 µm2; Threshold: 0.1 ; Maximum background intensity: 2. In order to classify detected cells into tumor cells, immune cells (TILs), stromal cells and others (false detections, background), we used neural network as a machine learning method with 8 hidden layers (maximum iterations: 100). The features used in the classification are listed below.<br>Nucleus: Area<br>Nucleus: Perimeter<br>Nucleus: Circularity<br>Nucleus: Max caliper<br>Nucleus: Min caliper<br>Nucleus: Eccentricity<br>Nucleus: Hematoxylin OD mean<br>Nucleus: Hematoxylin OD sum<br>Nucleus: Hematoxylin OD std dev<br>Nucleus: Hematoxylin OD max<br>Nucleus: Hematoxylin OD min<br>Nucleus: Hematoxylin OD range<br>Nucleus: Eosin OD mean<br>Nucleus: Eosin OD sum<br>Nucleus: Eosin OD std dev<br>Nucleus: Eosin OD max

Nucleus: Eosin OD min
Nucleus: Eosin OD range
Cell: Area
Cell: Perimeter
Cell: Circularity
Cell: Max caliper
Cell: Min caliper
Cell: Eccentricity
Cell: Eosin OD mean
Cell: Eosin OD std dev
Cell: Eosin OD max
Cell: Eosin OD min
Cytoplasm: Eosin OD mean
Cytoplasm: Eosin OD std dev
Cytoplasm: Eosin OD max
Cytoplasm: Eosin OD min
Nucleus/Cell area ratio
Smoothed: 25 µm: Nucleus: Area
Smoothed: 25 µm: Nucleus: Perimeter
Smoothed: 25 µm: Nucleus: Circularity
Smoothed: 25 µm: Nucleus: Max caliper
Smoothed: 25 µm: Nucleus: Min caliper
Smoothed: 25 µm: Nucleus: Eccentricity
Smoothed: 25 µm: Nucleus: Hematoxylin OD mean
Smoothed: 25 µm: Nucleus: Hematoxylin OD sum
Smoothed: 25 µm: Nucleus: Hematoxylin OD std dev
Smoothed: 25 µm: Nucleus: Hematoxylin OD max
Smoothed: 25 µm: Nucleus: Hematoxylin OD min
Smoothed: 25 µm: Nucleus: Hematoxylin OD range
Smoothed: 25 µm: Nucleus: Eosin OD mean
Smoothed: 25 µm: Nucleus: Eosin OD sum
Smoothed: 25 µm: Nucleus: Eosin OD std dev
Smoothed: 25 µm: Nucleus: Eosin OD max
Smoothed: 25 µm: Nucleus: Eosin OD min
Smoothed: 25 µm: Nucleus: Eosin OD range
Smoothed: 25 µm: Cell: Area
Smoothed: 25 µm: Cell: Perimeter
Smoothed: 25 µm: Cell: Circularity
Smoothed: 25 µm: Cell: Max caliper
Smoothed: 25 µm: Cell: Min caliper
Smoothed: 25 µm: Cell: Eccentricity
Smoothed: 25 µm: Cell: Eosin OD mean
Smoothed: 25 µm: Cell: Eosin OD std dev
Smoothed: 25 µm: Cell: Eosin OD max
Smoothed: 25 µm: Cell: Eosin OD min
Smoothed: 25 µm: Cytoplasm: Eosin OD mean
Smoothed: 25 µm: Cytoplasm: Eosin OD std dev
Smoothed: 25 µm: Cytoplasm: Eosin OD max
Smoothed: 25 µm: Cytoplasm: Eosin OD min
Smoothed: 25 µm: Nucleus/Cell area ratio
Smoothed: 25 µm: Nearby detection counts
Smoothed: 50 µm: Nucleus: Area
Smoothed: 50 µm: Nucleus: Perimeter
Smoothed: 50 µm: Nucleus: Circularity
Smoothed: 50 µm: Nucleus: Max caliper
Smoothed: 50 µm: Nucleus: Min caliper
Smoothed: 50 µm: Nucleus: Eccentricity
Smoothed: 50 µm: Nucleus: Hematoxylin OD mean
Smoothed: 50 µm: Nucleus: Hematoxylin OD sum
Smoothed: 50 µm: Nucleus: Hematoxylin OD std dev
Smoothed: 50 µm: Nucleus: Hematoxylin OD max
Smoothed: 50 µm: Nucleus: Hematoxylin OD min
Smoothed: 50 µm: Nucleus: Hematoxylin OD range
Smoothed: 50 µm: Nucleus: Eosin OD mean
Smoothed: 50 µm: Nucleus: Eosin OD sum
Smoothed: 50 µm: Nucleus: Eosin OD std dev
Smoothed: 50 µm: Nucleus: Eosin OD max
Smoothed: 50 µm: Nucleus: Eosin OD min

Smoothed: 50 µm: Nucleus: Eosin OD range
Smoothed: 50 µm: Cell: Area
Smoothed: 50 µm: Cell: Perimeter
Smoothed: 50 µm: Cell: Circularity
Smoothed: 50 µm: Cell: Max caliper
Smoothed: 50 µm: Cell: Min caliper
Smoothed: 50 µm: Cell: Eccentricity
Smoothed: 50 µm: Cell: Eosin OD mean
Smoothed: 50 µm: Cell: Eosin OD std dev
Smoothed: 50 µm: Cell: Eosin OD max
Smoothed: 50 µm: Cell: Eosin OD min
Smoothed: 50 µm: Cytoplasm: Eosin OD mean
Smoothed: 50 µm: Cytoplasm: Eosin OD std dev
Smoothed: 50 µm: Cytoplasm: Eosin OD max
Smoothed: 50 µm: Cytoplasm: Eosin OD min
Smoothed: 50 µm: Nucleus/Cell area ratio
Smoothed: 50 µm: Nearby detection counts
In order to help the algorithm perform an accurate classification, we also added smoothed object features at 25 µm and 50 µm radius to supplement the existing measurements of individual cells. Our algorithm (NN192) is available upon request and will be publicly available after publication.

For manuscripts utilizing custom algorithms or software that are central to the research but not yet described in published literature, software must be made available to editors/reviewers. We strongly encourage code deposition in a community repository (e.g. GitHub). See the Nature Research guidelines for submitting code & software for further information.

Data

Policy information about availability of data

All manuscripts must include a data availability statement. This statement should provide the following information, where applicable:

- Accession codes, unique identifiers, or web links for publicly available datasets
- A list of figures that have associated raw data
- A description of any restrictions on data availability

Provide your data availability statement here.

Field-specific reporting

Please select the one below that is the best fit for your research. If you are not sure, read the appropriate sections before making your selection.

☒ Life sciences ☐ Behavioural & social sciences ☐ Ecological, evolutionary & environmental sciences

For a reference copy of the document with all sections, see nature.com/documents/nr-reporting-summary-flat.pdf

Life sciences study design

All studies must disclose on these points even when the disclosure is negative.

Sample size	*Describe how sample size was determined, detailing any statistical methods used to predetermine sample size OR if no sample-size calculation was performed, describe how sample sizes were chosen and provide a rationale for why these sample sizes are sufficient.*
Data exclusions	*Describe any data exclusions. If no data were excluded from the analyses, state so OR if data were excluded, describe the exclusions and the rationale behind them, indicating whether exclusion criteria were pre-established.*
Replication	*Describe the measures taken to verify the reproducibility of the experimental findings. If all attempts at replication were successful, confirm this OR if there are any findings that were not replicated or cannot be reproduced, note this and describe why.*
Randomization	*Describe how samples/organisms/participants were allocated into experimental groups. If allocation was not random, describe how covariates were controlled OR if this is not relevant to your study, explain why.*
Blinding	*Describe whether the investigators were blinded to group allocation during data collection and/or analysis. If blinding was not possible, describe why OR explain why blinding was not relevant to your study.*

Reporting for specific materials, systems and methods

We require information from authors about some types of materials, experimental systems and methods used in many studies. Here, indicate whether each material, system or method listed is relevant to your study. If you are not sure if a list item applies to your research, read the appropriate section before selecting a response.

Materials & experimental systems

n/a	Involved in the study
☒	☐ Antibodies
☒	☐ Eukaryotic cell lines
☒	☐ Palaeontology
☒	☐ Animals and other organisms
☒	☐ Human research participants
☐	☒ Clinical data

Methods

n/a	Involved in the study
☒	☐ ChIP-seq
☒	☐ Flow cytometry
☒	☐ MRI-based neuroimaging

Clinical data

Policy information about clinical studies

All manuscripts should comply with the ICMJEguidelines for publication of clinical research and a completedCONSORT checklist must be included with all submissions.

Clinical trial registration	*Provide the trial registration number from ClinicalTrials.gov or an equivalent agency.*
Study protocol	*Note where the full trial protocol can be accessed OR if not available, explain why.*
Data collection	*Describe the settings and locales of data collection, noting the time periods of recruitment and data collection.*
Outcomes	*Describe how you pre-defined primary and secondary outcome measures and how you assessed these measures.*
